# Chromatographic Method for Evaluation of Polymeric GC Stationary Phases Ageing Using the Novel Non-Cross-Linked Poly(3-(Tributoxysilyl)Tricyclononene-7) as the Model Stationary Phase

**DOI:** 10.3390/polym13111899

**Published:** 2021-06-07

**Authors:** Anastasiia Kanateva, Maxim Bermeshev, Dmitrii Alentiev, Alexander A. Korolev, Alexander Kurganov

**Affiliations:** A.V. Topchiev Institute of Petrochemical Synthesis, Russian Academy of Science, 119991 Moscow, Russia; bmv@ips.ac.ru (M.B.); d.alentiev@ips.ac.ru (D.A.); akorolev@ips.ac.ru (A.A.K.); kurganov@ips.ac.ru (A.K.)

**Keywords:** norbornene polymer, polymer stationary phase, crosslinked polymer, ageing, thermodynamic parameters, sorption

## Abstract

The chromatographic properties and thermal stability are investigated for the polymeric stationary phase based on the norbornene polymer. It was shown that without additional cross-linking, poly(3-(tributoxysilyl)tricyclononene-7) demonstrates properties similar to liquid chromatographic stationary phases. It was also found to be more thermally stable than previously studied trimethylsilyl- and trimethoxysilyl- derivatives. The long-term heating at 170 °C resulted in an increase of mass transfer rate between stationary and mobile phases which could be observed as a decrease of parameter C of Van Deemter equation. This effect is rather unusual, as the polymeric stationary phases tend in decrease of the layer volume and porosity while ageing. Additionally, the values of thermodynamic parameters of sorption are calculated for the polymeric stationary phase: enthalpy of sorption varied −28 to −37 kJ/mol, entropy change was −41 to −51 J/mol K. The compensation curves were plotted for the alkanes, arenes, and alcohols, and the parameters of compensation plot were calculated, demonstrating the different sorption mechanisms both for hydrocarbons and oxygen-containing compounds, and different classes of organic compounds.

## 1. Introduction

Over the past decades, membrane technologies for the separation of vapors and gases have attracted more and more attention: they are used to remove carbon dioxide from flue gases, purify hydrogen in modern power plants, and many papers are devoted to the separation of light hydrocarbons. Polymeric membranes are among the most studied due to their high separation selectivity and relatively low cost. At the same time, the problem of scaling up while maintaining gas permeability and separation selectivity remains relevant, both upward when creating industrial plants, and downward when using membrane polymers as chromatographic stationary phases. Even microporous polymeric ultrathin membranes, actively studied recent years due to their high permeability, have a thickness of about 1 μm, which is 4–10 times larger than the thickness of the polymer layer in a chromatographic column. That is why utilization of membrane polymers as chromatographic stationary phases demands the devoted investigations of their properties.

**Figure 1:**
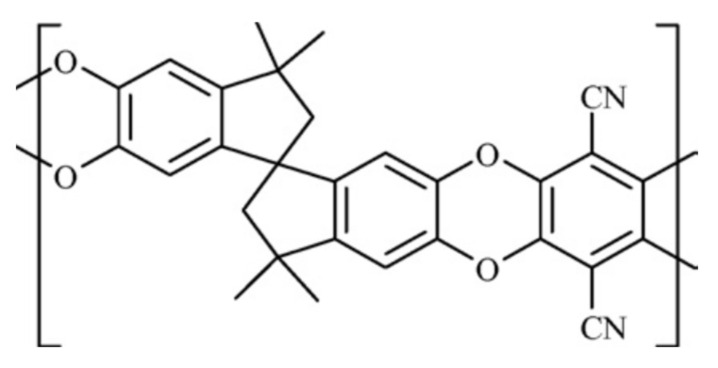
Structures of PIM-1 polymer. The detailed discussion of polymers with intrinsic porosity may be found in [10].

The most common approach supposes that the characteristics of the resulting thin polymer film/layer might be controlled by selecting organic monomers [1,2] and optimizing the conditions for film formation [3,4,5]. However, significant changes in polymer structure and even chemical composition can also occur during operation; therefore, the physical and chemical ageing of microporous polymers remain the important issue. In the case of physical ageing, gas chromatography (GC) may be used as a structurally sensitive method, which makes it possible to assess both the general chemical stability of the polymer, including stability at elevated temperatures, and the existence of individual phase transitions, i.e., changes in the physical state of the polymer layer. At the same time, the study of the ageing process itself using chromatographic methods has not yet been developed to the standard method. It is known that the transport of gases in thin polymer layers is often described using the gas permeability value P, which includes both the thermodynamic component (solubility coefficient S) and the kinetic component (diffusion coefficient D_S_). Solubility S is interpreted as a parameter proportional to Henry’s equilibrium constant, which means that changes in solubility should correlate with the solute retention coefficient k [6,7,8,9]. Despite the different previous histories of the samples used in chromatography and membrane measurements, different film thicknesses, and different testing procedures, chromatographic data can be used to refine the results obtained by other methods. For example, during the chromatographic investigation of polymeric stationary phases [10,11,12,13,14] of different natures with similar membrane properties an increase in the retention coefficient k was observed for several samples with sterically hindered structures of polymers such as PIM-1 (see Figure 1) after accelerated thermal ageing. This phenomenon indicates an increase in Henry’s constants and, therefore, in solubility of sorbates in the stationary phases. At the same time the thermodynamic parameters show that an increase in solubility occurs mainly due to decrease in the entropy of the process, while the enthalpy values remained almost unchanged. Therefore, thermal ageing induces rearrangements of polymer chains, creating more adapted sites for solutes retention and, in turn, changes the number of solute/stationary phase interactions.

**Figure 2:**
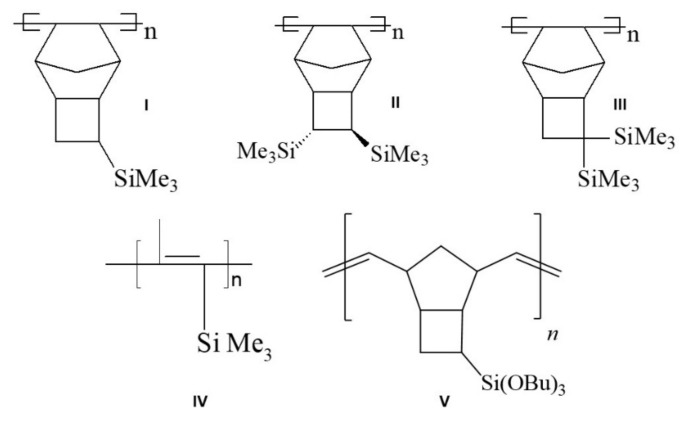
Structures of the investigated polymers: I—addition poly(3-trimethylsilyltricyclononene-7), II—addition poly(3,4-bis(trimethylsilyl)tricyclononene-7), III—addition poly(3,3-bis(trimethylsilyl)tricyclononene-7), IV—poly(trimethylsilylpropine), V—metathesis poly(3-(tributoxysilyl)tricyclononene-7).

The detailed studies were previously carried out on polyacetylenes (poly(trimethylsilylpropine) (PTMSP), Figure 2, structure IV) and polymers of the norbornene series [11,12,13] (Figure 2, structures I–III) using chromatographic method, when the polymers were used as the stationary phases in GC separations. Their chromatographic properties and thermal stability have been described in detail [14], and this work focuses on the study of a more sterically hindered polymer metathesis poly(3-(tributoxysilyl)tricyclononene-7) (PTBSN), the structure of which is also shown in Figure 2 (structure V).

As we could expect from the structure and preliminary membrane properties evaluation, the properties of PTBSN should be similar to those of other tricyclononene-7-based polymers. However, the experimental data showed that non-crosslinked PTBSN possessed rather low glass transition temperature, and in gas chromatographic conditions might demonstrate separation properties of “liquid” stationary phase, when separation is a result of different solubility of analytes in the stationary phase. “Liquid” and “solid” stationary phases usually demonstrate different ageing features and using PTBSN as the model stationary phase one has the promising opportunity of comparing the ageing process of non- and crosslinked stationary phase of the same chemical nature. Consequently, the aim of the present paper was presentation of the results of the first part of this complex work—the evaluation of thermal stability of the non-crosslinked PTBSN stationary phase using a gas chromatographic method.

## 2. Materials and Methods

The following chemicals and reagents were used in the investigation: benzene (≥99%), toluene (≥99%), *m*-xylene (≥98%), *p*-xylene (≥98%), *n*-hexane (≥99%), *n*-heptane (≥99%), *n*-octane (≥99%), butanone (≥98%), nitromethane (≥95%), pyridine (≥99%), and methylene chloride (≥99%) were purchased from Sigma-Aldrich catalogue (Merck KGaA, Darmstadt, Germany). Ethanol (≥95%) and *n*-Butanol (≥95%) were purchased from the local supplier (Dia-m, Moscow. Russia). Poly(3-(tributoxysilyl)tricyclononene-7) (PTBSN, structure V, Figure 2) was prepared according to methodology described in [15] (Scheme 4), [16] via ring opening metathesis polymerization using WCl_6_ in toluene catalyst in Institute of Petrochemical Synthesis, RAS, and was used as is. The primary investigation of properties showed that in GC separation conditions PTBSN had to be in visco-elastic state, as the glass transition temperature of this polymer was −32 °C (241 K). Such a low value of *T*_g_ was rather unexpected, as all the previously studied polymers—the derivatives of norbornene and tricyclononene-7—had *T*_g_ values higher than 100 °C, and even polynorbornene itself has *T*_g_ = 35 °C (308 K). The reason of such low glass transition temperature of PTBSN was possibly in the higher molar mass of side radicals compared to the previously studied monomers, and in increased polymer chain mobility. As a result, the polymeric stationary phase based on PTBSN had the chromatographic properties similar to those of the liquid stationary phases. Higher *T*_g_ values might be received after the polymer cross-linking, which will be the main goal of the next paper.

Preparation of polymer-coated capillary column was performed according to the following procedure. The study was carried out with quartz capillary (Technology Equipment Glass Structures LLC, Saratov, Russia) having an average inner diameter 0.165 mm. Capillary had an aluminum outer coating. Inner surface of the capillary was activated by consequent washing with 1% solution of HF, water, 25% solution of NH_3_, water, and methanol. After that the capillary was dried in a stream of helium at 150 °C. After completing the drying, the capillary was filled with the 0.5–0.9 % (*w*/*w*) solution of PTBSN in methylene chloride, and one end of the column was sealed. The column was dragged through the fore-heating system with the temperature 130 °C at a rate of 5 mm/min and ended up in a thermostat at 90 °C. After the end of the procedure, the column was purged with helium at 90 °C for 10 min. The polymeric film thickness *d_f_*, µm, was calculated using the empirical formula:

(1) $$d_{f}=2.5\cdot \omega_{polymer}\cdot d_{cap.},$$

where *ω_polymer_* is the polymer concentration, %; *d_cap._* is diameter of the capillary, mm.

All the chromatographic experiments were performed on a GC-2010 chromatograph (Shimadzu, Kyoto, Japan) equipped with a flame-ionization detector. Helium was used as a carrier gas. Peak width at half height was used for evaluation of column efficiency.

**Figure 3:**
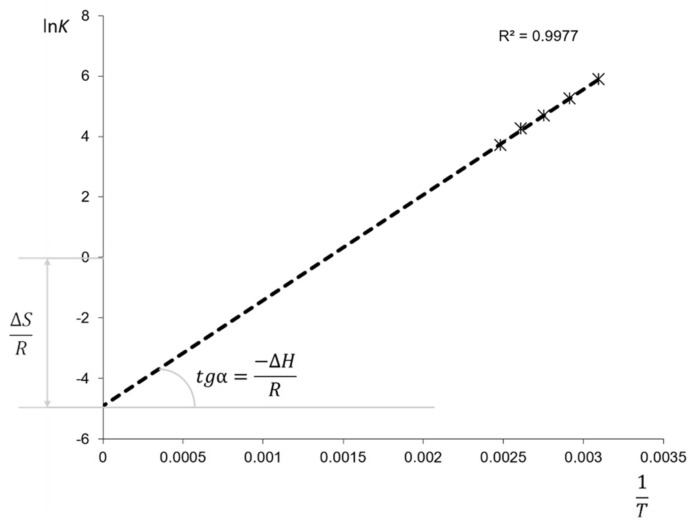
Van’t-Hoff dependence for benzene on PTBSN stationary phase. Carrier gas He, 70 kPa, temperatures 40, 60, 80, 100 and 120 °C.

The retention time of the sorbate in the chromatographic column depends on the Henry constant of the sorption equilibrium, so the thermodynamic constants of the sorption may be calculated from the retention data of the analytes at several different column temperatures using the Van’t-Hoff equation [17,18]. For this purpose, the retention factor *k* for each analyte was calculated at temperatures 40, 60, 80, 100 and 120 °C:

(2) $$k=\frac{t_{R}-t_{M}}{t_{M}},$$

where *t*_R_ is the retention time of the analyte at the definite temperature, and *t*_M_ is the elution time of the non-retained compound at the same temperature. Taking into consideration the phase ratio of the column, the logarithmic dependence of the distribution constant or retention factor on the inverse temperature was plotted:

(3) $$\Delta G=-RTlnK_{H}=\Delta H-T\Delta S$$

(4) $$lnK_{H}=lnk+lnb=\frac{-\Delta H}{TR}+\frac{\Delta S}{R}\;lnk=\frac{-\Delta H}{TR}+\frac{\Delta S}{R}-lnb$$

where ∆*H* is enthalpy of sorption, J/mol; *T*—temperature, K; *∆S* is entropy of sorption, J/mol K; *K_H_ = k·b*—distribution (Henry) constant; $b=\frac{V_{M}}{V_{S}}$ is the phase ratio of the column, *V_M_* and *V*_S_ are the mobile phase and polymer (stationary phase) volumes in the column; *R* = 8.314 J/mol K is the universal gas constant. The example of Van’t-Hoff dependence for benzene is shown in Figure 3.

The potential column efficiency was evaluated using Van Deemter curves [19], which are the semi-empirical dependence of the column efficiency either on the linear velocity of the carrier gas *u* or on the pressure drop on the column:

(5) $$H=A+\frac{B}{u}+C\cdot u,$$

where, *A*, *B*, and *C* are the coefficients characterizing the eddy diffusion in the mobile phase related to the non-ideal packing of the column (*A*), the longitudinal diffusion in the mobile phase resulting in peak broadening and dispersion of the analyte chromatographic zone (*B*), and the mass transfer kinetics on the phase boundary between mobile and stationary phases and in the volume of the stationary phase, i.e., inside the polymer layer (*C*, which is often considered as a sum of two coefficients *C_M_* and *C_S_*). *H* here is the height equivalent to the theoretical plate (HETP) characterizing the efficiency of separation: the lower is *H*, the higher is the column efficiency.

## 3. Results and Discussion

There is no consensus in the literature on the approaches to determining the thermal stability of chromatographic phases, but all researchers agree that with any method it is important to record such general properties of chromatographic stationary phases, as the values of potential (height equivalent to theoretical plate, HETP) and kinetic efficiency, selectivity, and thermodynamic parameters of sorption of compounds of various classes. To determine the thermal stability of the stationary phase, we used the following approaches:

1. to assess stability during temperature programming (short-term heating), the column was heated at a given temperature for 1 h, then cooled, and a model mixture of sorbates was separated under standard conditions (50 °C and 100 kPa). Then, the operation of heating the column was repeated by raising the heating temperature by 10–20 °C. Thus, the thermal stability of the column can be investigated over a rather wide temperature range. For each analyte, the retention time t_R_ and the column efficiency N (the number of theoretical plates) were determined, and Van Deemter curves were plotted, the thermodynamic parameters of sorption (enthalpy and entropy) were calculated;
2. to assess stability during long-term operation at high temperatures (isothermal mode), the column was heated at 170 and 200 °C for 7 h, then it was cooled, and the model mixture of sorbates was separated.

**Figure 4:**
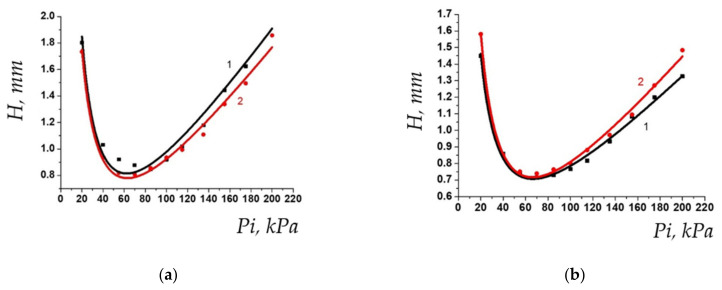
Van Deemter curves for the column based on PTBSN before (curve 1, black) and after (curve 2, red) heating cycles using method 1 for *n*-butane (**a**) and *n*-hexane (**b**).

Investigation of the properties of stationary phase based on polymer V showed that its chromatographic behavior corresponds to the behavior of non-porous liquid phases. Evaluation of thermal stability by the first method showed that short-term heating even in a wide temperature range does not significantly affect the properties of the stationary phase. Figure 4 demonstrates Van Deemter curves for *n*-butane and *n*-hexane, obtained before and after the cycle of heating the column from 50 to 200 °C. As can be seen from the figures, neither the position of the curve minimum, i.e., maximum potential column efficiency, nor slope of the right branch, i.e., the mass transfer rate (and hence the diffusion coefficients) change significantly, so the PTBSN-based chromatographic column occurs to have reasonable short-term thermal stability, which is required for GC stationary phases.

**Figure 5:**
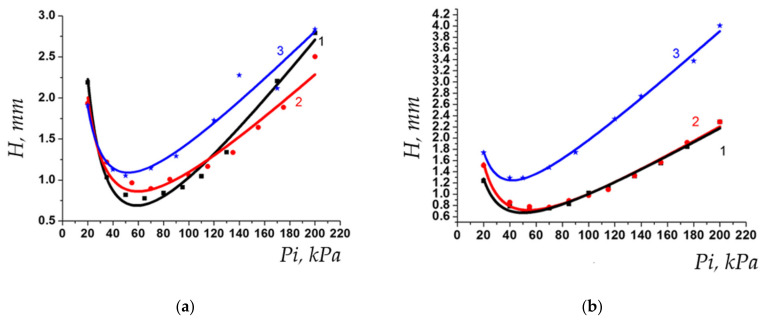
Changes in Van Deemter curve shape at accelerated thermal aging of PTBSN at 170 and 200 °C for *n*-butane (**a**) and *n*-hexane (**b**); 1—native polymer, 2—aging at 170 °C, 3—aging at 200 °C.

**Figure 6:**
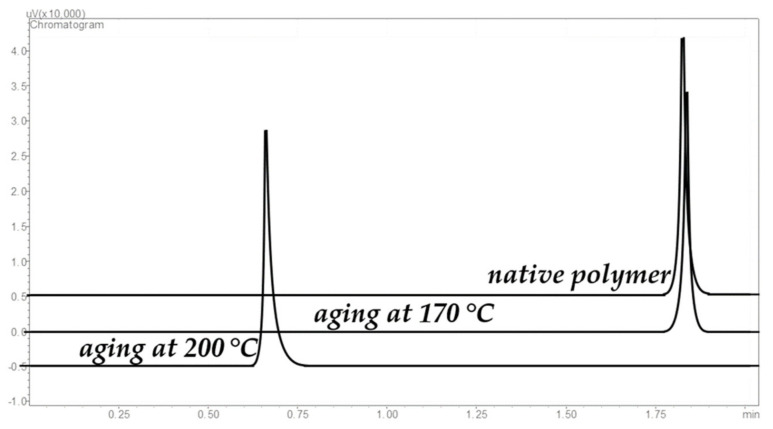
Changes in hexane retention on PTBSN stationary phase after thermal aging using method (2). Carrier gas helium, P_i_ 70 kPa, column temperature 130 °C, isotherm.

The thermal stability of the polymer was also evaluated in terms of long-term operation at elevated temperatures using method 2. Figure 5 demonstrates the change in the sorbent efficiency after accelerated thermal aging at 170 and 200 °C. The efficiency of the column did not change after the first heating cycle (21,000 theoretical plates/10 m column), but after the second one it dropped by at least 40% (Figure 5). The polymer turned out to be more thermally stable than its predecessors (structures I-III in Figure 2), and the separation of light hydrocarbons up to hexane takes less than 2 min. Prolonged heating at 170 °C resulted in a decrease in the value of the C coefficient of the Van Deemter equation for light hydrocarbons (up to butane), which indicates an acceleration of mass transfer between the mobile and stationary phases. At the same time, there are practically no changes for hexane after heating at 170 °C, but there is a significant deterioration in the kinetics of mass transfer after heating to 200 °C (Figure 5) and almost twice decrease of retention. Additionally, peak asymmetry increased after second round of heating, and peak tailing was observed (see Figure 6). This supposes realization of the non-linear equilibrium isotherm which is described by the Langmuir equation instead of linear Henry isotherm for the native polymer. The reason of such transition might be in the decrease of the quantity of the sites for solutes retention in the polymer structure. It is worth noting that consideration of the polymer as a “liquid” stationary phase does not exclude the possibility of sorption interactions between the polymer and the analyzed sorbates because of high molar mass of the polymer, which means that the adsorption theory might be applied to the polymer–sorbate system, which assumes the competitive adsorption of analytes and the appearance of nonlinear isotherms of the process in the presence of a limited number of sorption centers and a rather large coverage. Heating the polymer leads to the reorganization of its structure and, therefore, to decrease in the number of interaction centers in its structure, and thus increases the possibility of competitive processes and leads to the formation of a concave sorption isotherm described by the Langmuir equation.

Table 1, Table 2 and Table 3 show the obtained values of the thermodynamic functions of sorption for organic compounds of various classes for a column with a stationary phase based on PTBSN in the initial state and after two heating cycles at 170 and 200 °C. The calculated values of the enthalpy of sorption of hydrocarbons on the initial polymer varied for hydrocarbons in the range from −28 to −37 kJ/mol and practically did not change after both stages of heating. In contrast to hydrocarbons, the ∆*H* values for the studied alcohols increased after each stage. The value of the entropy loss ∆S for hydrocarbons also practically did not change and ranged from −41 to −51 J/(mol K) for the initial polymer, while for alcohols it increased, and for ethanol, practically threefold from −33 to −97 J/(mol K) (Table 1, Table 2 and Table 3). This may be due to the partial destruction of the polymer chain because of thermal degradation with the formation of polar groups in the polymer structure. Due to partial elimination of butoxy groups on the polymer chain, access to hydroxyl groups can appear, which results both in an increase in the retention of alcohols (this confirmed by experimental data), and in an increase in the values of entropy loss for polar compounds.

Such differences in the retention of hydrocarbons and alcohols are primarily associated with different mechanisms of sorption of these compounds. The similarity in the mechanisms of sorption for different classes of compounds can be estimated using compensation curves [12,13] ∆*H* = β∆*S* + α, where β is the compensation temperature (K).

**Figure 7:**
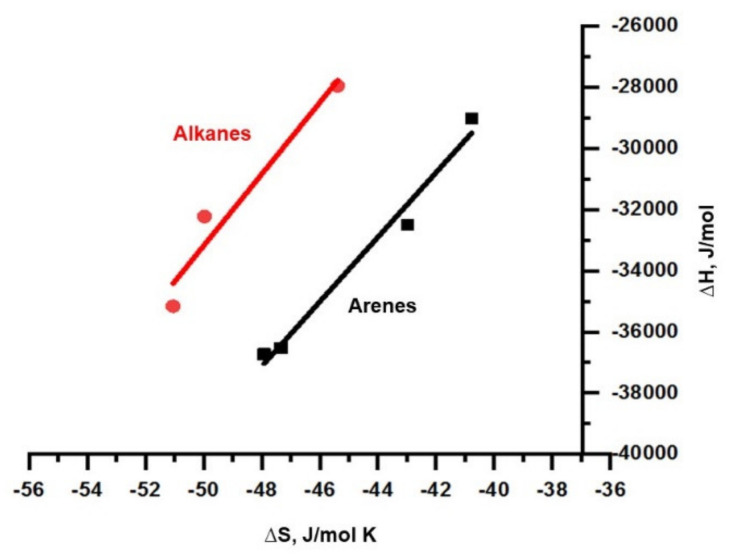
Compensation curves for poly(3-(tributoxysilyl)tricyclononene-7) (PTBSN) in the initial state for homologues series of *n*-alkanes and arenes.

Compensation curves for homologous series of alkanes and arenes on PTBSN are shown in Figure 7, and the values of the parameters of the compensation equation for alkanes, arenes, and alcohols are shown in Table 4. The difference in the compensation temperature β for hydrocarbons and alcohols is quite predictable, however, for homologous series alkanes and arenes, the difference in β values was also more than 100 K, which indicates differences in the mechanisms of sorption for these two classes.

**Figure 8:**
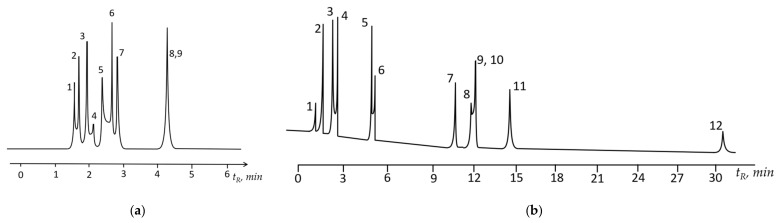
Separation of model mixtures on PTBSN stationary phase column in the initial state. (**a**) 1—ethanol, 2—n-hexane, 3—benzene, 4—*n*-butanol, 5—nitromethane, 6—*n*-octane, 7—toluene, 8—*m*-xylene, 9—*p*-xylene. Carrier gas helium, P_i_ 70 kPa, column temperature 130 °C, isotherm; (**b**) 1—*n*-pentane, 2—*n*-hexane, 3—benzene, 4—*n*-heptane, 5—toluene, 6—*n*-octane, 7—ethylbenzene, 8—*p*-xylene, 9—*m*-xylene, 10—*n*-nonane, 11—*o*-xylene, 12—*n*-decane. Carrier gas helium, P_i_ 120 kPa, column temperature 40 °C, isotherm.

To clarify the mechanism of sorbate-polymer interaction, Rohrschneider constants were calculated for the investigated stationary phase, which made it possible to characterize the contribution of various interactions to the retention of sorbates on the polymer stationary phase (see Table 5). As one can see from the table, the greatest contribution to retention is by orientational and donor-acceptor interactions (model compounds—nitromethane and pyridine), as well as the formation of hydrogen bonds with electron-donor groups of the stationary phase. This means that polar compounds capable of formation of hydrogen bonds might have larger retention on the studied stationary phase, and the stationary phase might be suitable for fast separations of polar compounds and aromatic hydrocarbons at elevated temperatures. The example of such separation is showed in Figure 8a, where eight sorbates are separated in less than 5 min at 130 °C.

This also means that the discussed stationary phase should have the isomeric selectivity, which is usually tested via separation of meta- and para-xylenes. From this point of view, the most prominent stationary phases for *m*-/*p*- isomers separation are the liquid crystals, having α values for this couple of analytes 1.14–1.21. Testing PTBSN stationary phase showed that it possesses the α = 1.09 and might be also utilized for separation of position isomers of xylene. The example of such separation is shown in Figure 8b, where compounds 8 and 9 are *m*- and *p*-xylenes, which were separated in the isothermal mode at 40 °C.

## 4. Conclusions

The chromatographic behavior of poly (3-(tributoxysilyl)tricyclononene-7) corresponds to the behavior of non-porous liquid phases. The investigation of thermal stability showed that short-term heating, even in a wide temperature range, does not significantly affect the properties of the stationary phase. Together with rather short separation times for hydrocarbons and alcohols, the PTBSN stationary phase may be considered as promising in the areas demanding fast separations at elevated temperatures, such as petroleum exploration and industrial gases control. Further prospects for this work will be associated with the preparation of a crosslinked stationary phase, since crosslinking traditionally additionally increases the thermal stability of the polymer. When applied to the investigated in the present paper stationary phase, crosslinking can also lead to a dramatic change in properties, as it will lead to the transition of the stationary phase from the category of liquid stationary phases to the category of solid polymer sorbents.

The difference in the compensation temperature β for hydrocarbons and alcohols and for the homologous series of alkanes and arenes indicates differences in the mechanisms of sorption for these classes. The largest contribution to retention is made by orientational and donor-acceptor interactions (model compounds—nitromethane and pyridine), as well as the formation of hydrogen bonds with electron-donor groups of the stationary phase (model compounds—ethanol and pyridine). Thermal aging of the polymer resulted in overall decrease in retention, while the selectivity of the polymeric stationary phase did not change significantly. This may be due to a decrease in the availability of pore space in the polymer structure, and not to thermal degradation of the stationary phase material.

The developed method for evaluation of thermal stability of the gas chromatographic stationary phase seems to be viable and allows comparison of the different states of the polymeric material in the GC capillary.

## Figures and Tables

**Table 1:** Thermodynamic parameters of sorption of hydrocarbons and alcohols in PTBSN in the initial state.

Sorbate	∆*H*, kJ/mol	∆*S*, J/(mol K)
Benzene	−29.0 ± 0.8	−41 ± 2
Toluene	−32.5 ± 0.3	−43.0 ± 0.7
*m*-Xylene	−36.7 ± 0.3	−47.9 ± 0.8
*p*-Xylene	−36.5 ± 0.4	−47 ± 1
*n*-Hexane	−28 ± 2	−45 ± 4
*n*-Heptane	−32.2 ± 0.5	−50 ± 1
*n*-Octane	−35.2 ± 0.4	−51 ± 1
*n*-Butanol	−34.4 ± 0.1	−55.1 ± 0.4
Ethanol	−21.4 ± 0.2	−33.3 ± 0.5

**Table 2:** Thermodynamic parameters of sorption of hydrocarbons and alcohols in PTBSN after the first stage of heating (170 °C).

Sorbate	∆*H*, kJ/mol	∆*S*, J/(mol K)
Benzene	−29 ± 1	−41 ± 3
Toluene	−32.62 ± 0.05	−43.6 ± 0.1
*m*-Xylene	−36.7 ± 0.6	−48 ± 2
*p*-Xylene	−36.7 ± 0.7	−48 ± 2
*n*-Hexane	−28 ± 1	−47 ± 4
*n*-Heptane	−31.4 ± 0.2	−47.5 ± 0.5
*n*-Octane	−35.4 ± 0.1	−52.0 ± 0.3
*n*-Butanol	−33 ± 2	−50 ± 6
Ethanol	−30 ± 5	−57 ± 4

**Table 3:** Thermodynamic parameters of sorption of hydrocarbons and alcohols in PTBSN after the second stage of heating (200 °C).

Sorbate	∆*H*, kJ/mol	∆*S*, J/(mol K)
Benzene	−33.0 ± 0.2	−52.6 ± 0.7
Toluene	−38.3 ± 0.1	−60.5 ± 0.3
*m*-Xylene	−43.1 ± 0.5	−67 ± 2
*p*-Xylene	−28 ± 2	−47 ± 6
*n*-Hexane	−38.0 ± 0.9	−69 ± 3
*n*-Heptane	−40 ± 1	−68 ± 3
*n*-Octane	−39.6 ± 0.5	−64 ± 2
*n*-Butanol	−43 ± 3	−87 ± 5
Ethanol	−39 ± 2	−97 ± 5

**Table 4:** Parameters of the compensation equation ∆*H* = β∆*S* + α for homologues series of n-alkanes, and alcohols for poly(3-(tributoxysilyl)tricyclononene-7) (PTBSN) in the native state and after ageing at 170 and 200 °C.

Homologues Series	Compensation Temperature β, K			Constant Term α, J/mol		
	Native	170 °C	200 °C	Native	170 °C	200 °C
*n*-alkanes	1173	1246	508	25,488	29,054	−4203
arenes	1057	1081	692	13,590	15,042	3359
alcohols	596	286	147	15,420	18,614	–30,139

**Table 5:** Model sorbates of Rohrschneider model and their retention on PTBSN in the initial state.

Model Compounds	Sorbent Selectivity Constants	The Modelled Groups of the Compounds	Polymer-Sorbate Interaction	$t_{R}^{\prime },\;\min$	Constants Values
Benzene	$X=\frac{I_{benzene}^{polymer}-I_{benzene}^{squalane}}{100}$ $I_{benzene}^{squalane}=$ 649	Aromatic and non-saturated hydrocarbons	π-complexation	0.97	1.15
Ethanol	$Y=\frac{I_{ethanol}^{polymer}-I_{ethanol}^{squalane}}{100}$ $I_{ethanol}^{squalane}=$ 384	Alcohols, primary and secondary amines, fatty acids, ex. lower acids	Hydrogen bonding with the electron-donor groups of the polymer	0.38	2.02
Butanone	$Z=\frac{I_{butanone}^{polymer}-I_{butanone}^{squalane}}{100}$ $I_{butanone}^{squalane}=$ 531	Ketones, aldehydes, asters and ethers, FAME	Donor-acceptor complexation	0.65	1.69
Nitromethane	$U=\frac{I_{nitromethane}^{polymer}-I_{nitromethane}^{squalane}}{100}$ $I_{nitromethane}^{squalane}=$ 457	Nitro-, nilril-compounds, halogen derivatives of aromatic hydrocarbons,	Orientational and donor-acceptor complexation	1.15	3.35
Pyridine	$S=\frac{I_{pyridine}^{polymer}-I_{pyridine}^{squalane}}{100}$ $I_{pyridine}^{squalane}=$ 695	Aromatic amines, pyridines, heterocyclic basic compounds	Hydrogen bonding with the electron-donor groups of the polymer, donor-acceptor complexation	10.84	3.89

## Data Availability

Not applicable.
